# Secretory Carcinoma of Male Breast: Case Report and Review of the Literature

**DOI:** 10.4061/2011/704657

**Published:** 2011-02-06

**Authors:** S. Gabal, S. Talaat

**Affiliations:** Department of Pathology, Faculty of Medicine, Cairo University, Giza 12655, Egypt

## Abstract

Secretory carcinoma is a rare low-grade breast carcinoma, initially termed “juvenile breast cancer,” but it is now known to occur in adults of both sexes. It is the only epithelial tumor of the breast with a balanced translocation, t(12;15), that creates an ETV6-NTRK3 gene translocation. In this paper, a 19-year-old male patient has had a right breast mass for 9 years which suddenly increased in size with no evidence of palpable axillary lymph nodes. The mass was excised for frozen section and was diagnosed as malignant growth for simple mastectomy. Microscopic examination revealed the classical features of secretory carcinoma. The tumor cells were positive for EMA and S-100 protein and focally positive for cytokeratin and ER but negative for progesterone receptor, CD34, and CEA. Four months later the patient developed ipsilateral axillary lymph node enlargement, with lymph node metastases in five of the dissected 19 lymph nodes. The patient was treated with six courses of chemotherapy and radiotherapy. *Conclusion*. Though considered an indolent neoplasm, secretory carcinoma does metastasize to lymph nodes. Surgery in the form of mastectomy with axillary clearance is the treatment of choice. This paper includes a rare case report of secretory carcinoma in young male patient, with axillary lymph node metastasis in spite of the indolent nature that this tumor is known to display.

## 1. Background

Secretory carcinoma is a rare (<1%) low-grade breast carcinoma. it is most common under the age of 30, and it is the most common type of breast carcinoma in children [1]. This entity was initially termed “Juvenile breast cancer" by McDivitt and Stewart, based on the fact that the average age of the seven patients described in their series was nine years old with a range of three to fifteen years [2]. Although originally described in children, it is now known to occur in adults of both sexes [3], and male to female ratio is 1 : 6 [4]. The tumor is the only epithelial tumor of the breast with a balanced translocation, t(12;15), that creates an ETV6-NTRK3 gene translocation [3]. The biological consequence of this translocation is the fusion of the dimerization domain of a transcriptional regulator (ETV6) with a membrane receptor tyrosine kinase (NTRK3) that activates the Ras-Mek1 and PI3K-Akt pathways which are important for breast cell proliferation and survival. This specific translocation is associated with congenital fibrosarcoma and mesoblastic nephroma, two morphologically similar pediatric mesenchymal tumors with no epithelial features [7].

The diagnostic microscopic criteria are abundant granular cytoplasm or clear vacuolated cytoplasm. Tubule formation is common and may have secretion in the lumens. Follicular pattern (thyroid-like) might be seen. The secretory material in cells, lumens, and stroma is mucicarmine, alcian blue, and PAS positive, diastase resistant. Fibrous bands are often prominent. It is characterized by low-grade nuclear cytology, bland, uniform nuclei, and rare mitotic figures. Sheet-like growth with mainly circumscribed margins with occasional foci of infiltration and in situ component are common [1].

Very few authors have performed immunohistochemical studies on secretory carcinomas of male breasts. These tumors are said to be epithelial membrane antigen, cytokeratin, carcinoembryonic antigen (polyclonal), S-100, and *α* lactalbumin positive [8].

Though considered an indolent neoplasm, secretory carcinoma does metastasize to lymph nodes and recur after local excision [9]. Tavassoli and Norris suggested three features of secretory carcinoma that indicate a favorable prognosis: tumor size less than 2 cm, age of less than 20 years at diagnosis, and tumor with circumscribed margins [10]. De Bree found that secretory carcinomas in men appear to be more aggressive [11].

Surgery in the form of mastectomy with axillary clearance is the treatment of choice [9]. There are several reported cases of patients with secretory breast carcinoma with distant metastases who were treated with either single agent or combination chemotherapy without success. Among the drugs reported are 5-FU, vindesine, mitomycin, prednisone, adriamycin, epirubicin, cyclophosphamide, carboplatin, and even newer active agents such as docetaxel. These data clearly show that this neoplasm is not chemosensitive as all of the patients treated with chemotherapy showed disease progression while on treatment [4].

## 2. Case Report

A 19-year-old male patient has had a right breast mass for 9 years which suddenly increased in size with no evidence of palpable axillary lymph nodes. Serum tumor markers and other routine blood test were normal. The liver ultrasonography, chest X-ray, and bone scan were negative for metastases. The mass was excised for frozen section and was diagnosed as malignant growth for simple mastectomy. 

**Figure 1:**
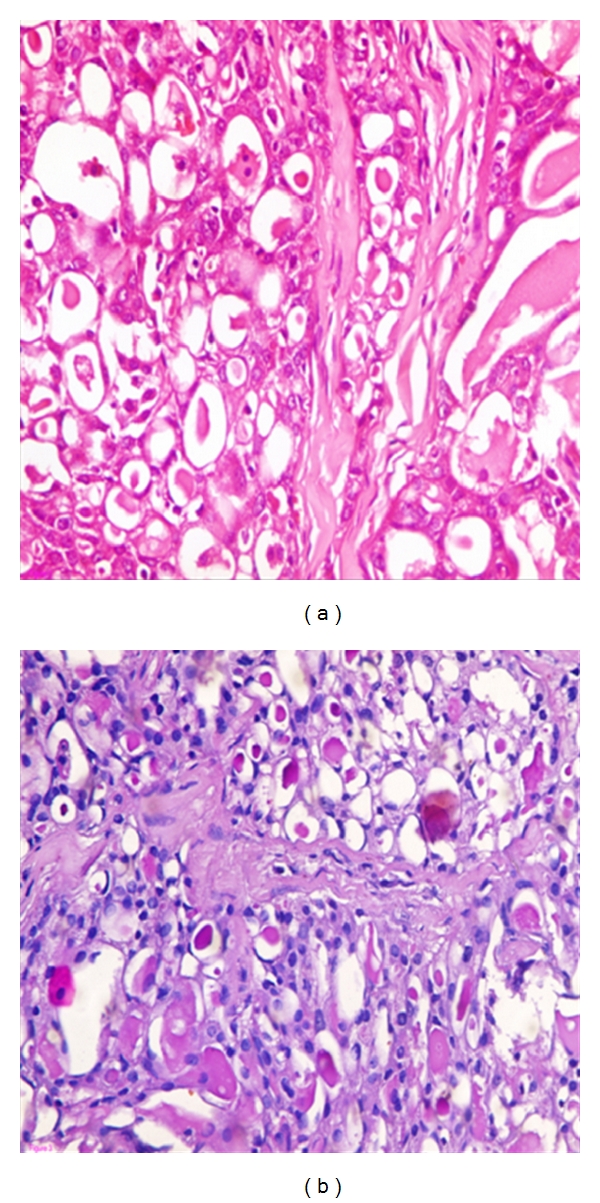
Secretory carcinoma shows microcystic pattern and intraluminal secretion, (a) (H&E) and (b) (PAS).

**Figure 2:**
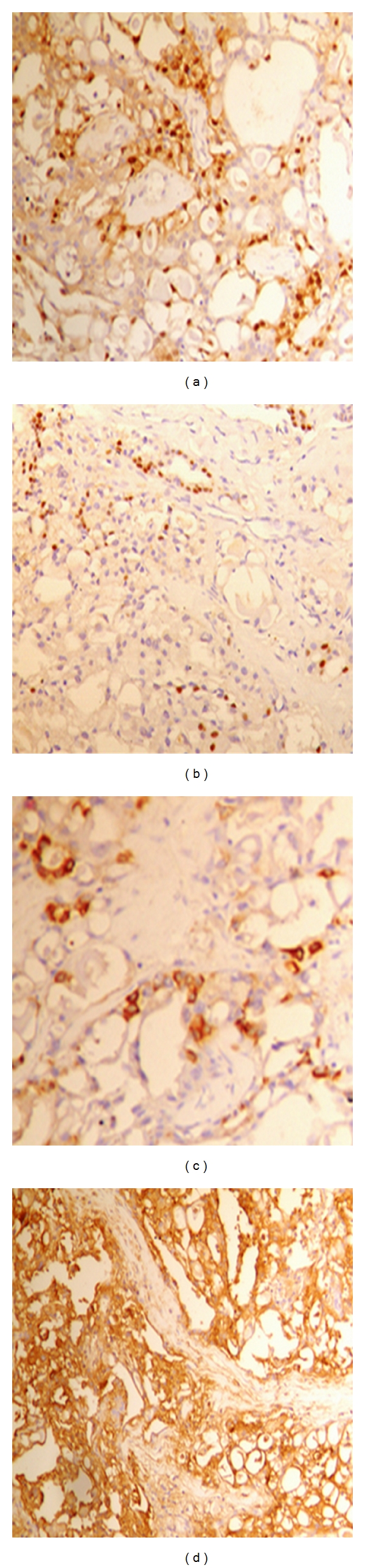
The tumor cells show positive nuclear and cytoplasmic staining by S100 antibody (a) positive cytoplasmic staining by EMA antibody, (b) positive cytoplasmic staining by pan cytokeratin, (c) and some showed positive nuclear staining by ER antibody (d).

Grossly, the mass was circumscribed, 2 × 2 cm, firm, and had white glistening cut section. Microscopic examination revealed the classical features of secretory carcinoma with a microcystic pattern (Figure 1(a)) with abundant intra and extracellular PAS-positive secretory material (Figure 1(b)). The tumor cells were mostly bland looking with round or oval nuclei and had few scattered nucleoli. No tumor infiltration was present at the nipple or at surgical margins. On immunohistochemistry, the tumor cells were positive for EMA and S-100 protein and focally positive for cytokeratine and ER (Figures 2(a), 2(b), 2(c), and 2(d)) but negative for progesterone receptor, CD34, and CEA (Dako, Carpinteria, CA, USA).

Four months later the patient developed ipsilateral axillary lymph nodes enlargement which when biopsied revealed lymph node metastasis of the previously excised secretory carcinoma. Then the patient underwent axillary evacuation. On microscopic examination, lymph node metastases were detected in five of the dissected 19 lymph nodes. Then it was decided for the patient to be treated with six courses of chemotherapy and radiotherapy.

## 3. Discussion

Secretory carcinoma is a very rare type of breast carcinoma. Tavassoli and Norris [10] reported 4 cases of SC in a retrospective series of 7038 breast carcinoma cases, and De Bree et al. [11] found one case of SC among 3000 breast carcinoma cases.

The age at presentation varies from 3 to 66 years with a median age of 23 years [8]. The case presented herein is extremely unusual as secretory carcinoma, particularly metastatic secretory carcinoma, has rarely been reported in males. Literature search identified only 18 other cases in males. Our case was older than the average age reported for secretory carcinoma in males which is 17 years. Only the case reported by Kuwabara was in old age (66 years) [9].  Table 1 summarizes the main clinical features of the cases of secretory carcinoma reported in males. 

Secretory carcinomas can demonstrate several histological patterns including, solid, microcystic, and ductal, with many tumors containing all three patterns [25]. The tumor cells are polygonal with granular eosinophilic cytoplasm, with intracellular and extracellular PAS- and alcian-blue-positive secretions [25]. Atypia is minimal or absent and mitotic activity is low [26]. A study has reported that only 4 and 2 out of 13 cases expressed estrogen and progesterone receptor, respectively, and only two were HER2 positive [27]. In the current case, the tumor had positive ER and negative progesterone receptors.

The most frequent clinical presentation is of an asymptomatic mobile mass, which is usually subareolar. The tumor size varies from 1 cm to 16 cm with an average diameter of 3 cm [28]. Our patient had a mass of 2 × 2 cm. As the patient reported that the lesion had been present for at least 9 years, it is assumed that it had behaved in a slow growing, indolent fashion. This is supported by other reported cases [25]. In this regard, Herz et al. have reported a MIB1 labeling index of 11.4% (range: <1 to 34%) [26].

Surgery is considered the primary treatment of secretory carcinoma; however, due to scarcity of reported cases, no published guidelines for surgical management exist. However, few studies reported local recurrence in some patients therefore, mastectomy appears to be a sound surgical choice [1, 11, 25, 29]. There are no data, however, on conservative treatment, but this option could be explored particularly in cases where breast development has not yet occurred. In regard to the management of the axilla, the overall incidence of axillary lymph node infiltration is around 30% in children and adults regardless of gender [28]; hence, axillary lymph node dissection is advocated by some authors for tumors ≥2 cm [25]. Nevertheless, sentinel node biopsy, may be useful for secretory carcinomas. A recent report on a 9-year-old girl treated with simple mastectomy and axillary sentinel lymph node biopsy shows that this is feasible [29].

Postoperative radiotherapy [28] and adjuvant chemotherapy [10] have been used on at least two occasions. There is at present insufficient evidence to recommend either approach in the management of secretory carcinoma.

Local recurrence after a long disease-free interval has been described in numerous cases; [1, 8, 12, 30], however these occurred in patients that underwent conservative surgery. Distant metastases from secretory carcinoma are extremely rare with only four cases reported [4]. Another reported patient remained disease free at a follow-up of 13 months despite having 12 out of 14 positive nodes and not having received adjuvant chemotherapy [11].

## 4. Conclusion

Secretory carcinoma is a rare slow-growing tumor, and though considered an indolent neoplasm, it does metastasise to lymph nodes. Surgery in the form of mastectomy with axillary clearance is the treatment of choice.

##  Authors' Contributions

Both authors equally contributed to the diagnosis of the patient and the preparation of the manuscript for publication. Samia Gabal wrote the manuscript and did the pathological analysis and discussion. Sahar Talaat critically reviewed the manuscript.

## Figures and Tables

**Table 1:** Data on 18 males with secretory breast cancer [3].

Author	Year	Age	Duration of symptoms	Size (cm)	Axillary status	Treatment	Hormone receptors	ETV6-NTRK3	Followup
Simpson and Barson [5]	1969	5	ND	ND	− (clinical)	LE	NE	NE	NED 4 y
Tavassoli and Norris [10]	1980	9	ND	ND	− (clinical)	LE	NE	NE	NED 1.75 y
Karl et al. [12]	1985	3	1 mo	1.5	+ (1/4)	SM+ALNS	NE	NE	ND
Roth et al. [13]	1988	23	21 years	2.0	− (0/21)	MRM	NE	NE	NED 4 y
Krausz et al. [14]	1989	24	Many years	4.0	ND	SM + RT (axilla)	NE	NE	DOD 20 y
Serour et al. [15]	1992	17	4 years	1.5	− (0/3)	WLE + ALND	ER − PR+	NE	NED 5 y
Lamovec and Bracko [16]	1994	20	ND	1.2	− (0/?)	MRM	ER+ PR+	NE	NED 1 y
Pohar-Marinsek and Golouh [6]	1994	20	6-7 years	1.2	− (clinical)	SM	ER+ PR+	NE	NED 6 m
Kuwabara et al. [17]	1988	66	3 years	3.0	+ (2/?)	MRM	ER − PR+	NE	NED 8 m
Vesoulis and Kashkari [18]	1998	33	10 years	1.5	ND	MRM	ER+ PR+	NE	ND
Kameyama et al. [19]	1998	50	ND	3.0	− (0/?)	MRM	ER+	NE	ND
Chevallier et al. [20]	1999	9	14 m	2.0	− (0/?)	LE + ALND	ER − PR−	NE	NED 45 m
Yildirim et al. [21]	1999	11	1 year	1.5	+ (1/18)	MRT + CT+ RT	ER−	NE	NED 12 m
Bhagwandeen and Fenton [22]	1999-2000	9	1 m	1.2	− (0/15)	MRM	ER − PR−	NE	NED 20 m
De Bree et al. [11]	2001	17	2 years	2.0	− (0–14)	MRM	ER − PR−	NE	NED 9 m
Niveditha et al. [23]	2004	19	2 years		ND	WLE	ER − PR−	NE	ND
Grabellus et al. [24]	2005	46 male-female transexual	ND	4.0	ND	LE	ER − PR−	PRESENT	ND
McDivitt and Stewart [2]	2005	52	10 years	7	+ 2/24	MRM + CT	ER − PR−	PRESENT	AWD 25 m
This case	2010	19	9	2	+ 5/19	SM + ALND	ER + PR−	NE	AWD

ND: not defined, LE: local excision, MRM: modified radical mastectomy, CT: chemotherapy, RT radiotherapy, NE: not examined, NED: not evidence of disease, AWD: alive with disease, ER: estrogen receptor, PR: progesterone receptor, SM: simple mastectomy, ALNS: axillary lymph node sampling, ALND: axillary lymph node dissection, WLE: wide local excision, DOD: died of disease.
